# Progresses in Fluorescence Imaging Guidance for Bone and Soft Tissue Sarcoma Surgery

**DOI:** 10.3389/fonc.2022.879697

**Published:** 2022-07-04

**Authors:** Zhenyi Chen, Huayi Huang, Siyuan He, Yi Wang, Lin Cai, Yuanlong Xie

**Affiliations:** Department of Orthopedics, Zhongnan Hospital, Wuhan University, Wuhan, China

**Keywords:** fluorescence imaging, bone sarcoma, soft tissue sarcoma, fluorescent probes, cancer imaging

## Abstract

R0 surgical resection is the preferred treatment for bone and soft tissue sarcoma. However, there is still a lack of precise technology that can visualize bone and soft tissue sarcoma during surgery to assist the surgeon in judging the tumor surgical boundary. Fluorescence imaging technology has been used in the diagnosis of cancer. It is a simple and essentially safe technique that takes no additional time during the operation. Intraoperative fluorescence imaging has potential application prospects in assisting the surgeons in judging the tumor boundary and improving the accuracy of surgical resection. This review mainly starts with clinical studies, animal experimentation, and newly designed probes of intraoperative fluorescence imaging of bone and soft tissue sarcoma, to appraise the application prospects of fluorescence imaging technology in bone and soft tissue sarcoma.

## Introduction

Traditionally, surgeons mainly use preoperative CT(computed tomography) and MRI(magnetic resonance imaging) to assess the tumor boundary within the surgeons’ naked eyes to select the scope of resection during the surgery (1). CT and MRI cannot be used in real-time and have limited tumor specificity (2). Assessments of the resection boundary based on the surgeons’ naked eyes are inaccurate and rely on surgeons’ experience. The intraoperative frozen section reduces surgical efficiency because of the unavoidable extension of the surgery period (3). It is urgent to find an auxiliary examination during the operation to judge the boundary between the tumor tissue and the normal tissue, which can improve the accuracy of the operation together with assistance in finding the tumor satellite foci (4).

The essence of intraoperative tumor fluorescence imaging is to allow fluorescent dyes to accumulate in tumor tissue during the operation so that the surgeons can find the boundary of the tumor. Fluorescent probes have different principles, such as EPR effects (enhanced permeability and retention) and antigen-antibody reactions. To date, fluorescence imaging has exhibited promising advantages in various tumors, such as brain tumors (5), breast cancer (6), and gastric cancer (7).

In recent years, intraoperative fluorescence imaging of bone and soft tissue sarcoma has been explored in clinical studies, animal experimentation, and these studies have led to the development of new probes.

## Principle of Fluorescence Imaging

### Non-Specific Fluorescent Probes

**Figure 1:**
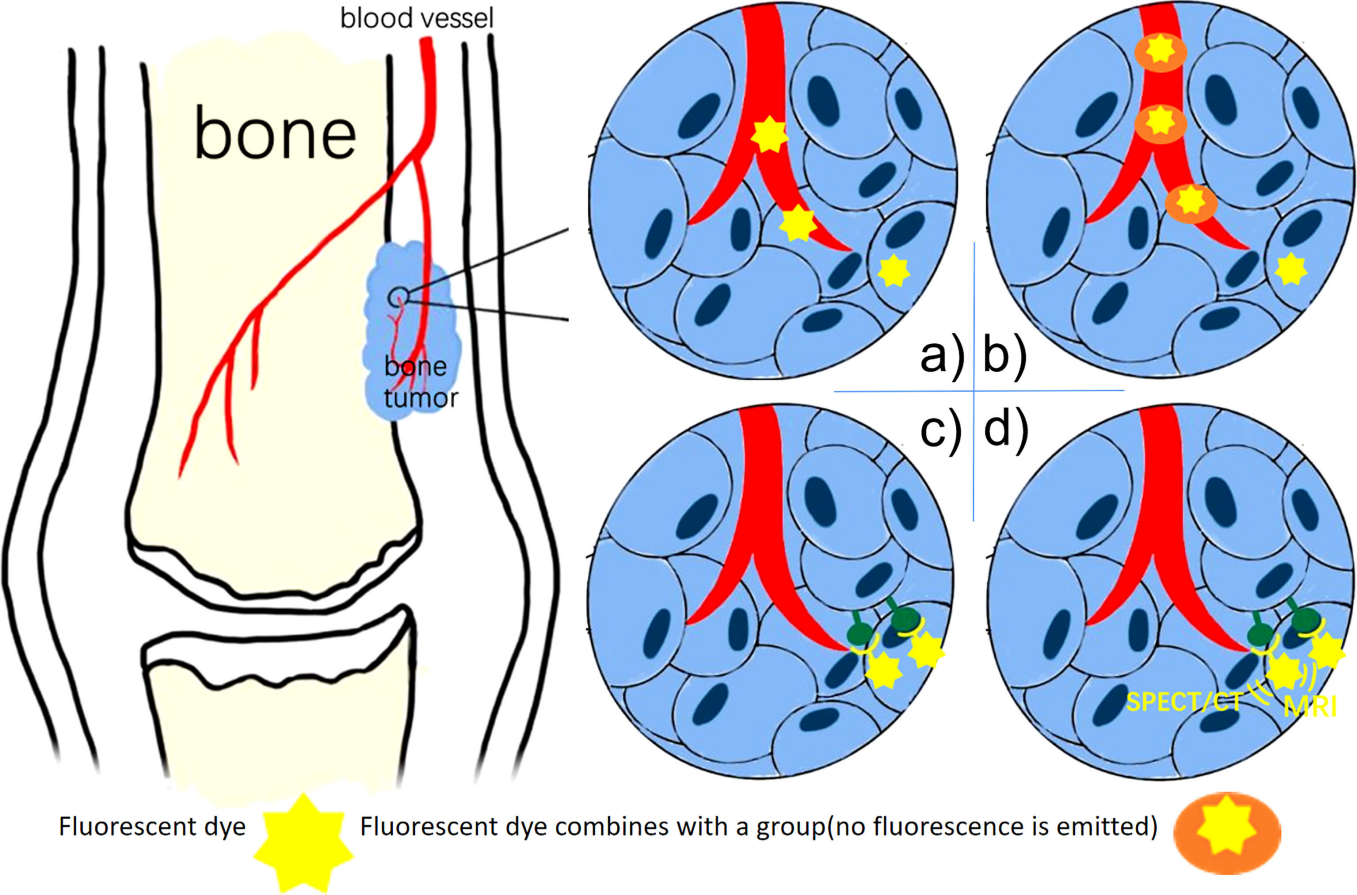
**(A)** Non-Specific Fluorescent Probes (ICG): the fluorescent dye remains in areas where the vasculature is highly disordered. **(B)** Activatable Fluorescence Probes: the dye fluorescence only when the group breaks down under tumor circumstances. **(C)** Specific fluorescent Probes: the fluorescent dye combines antibodies bind to biomarkers. **(D)** Multimodality Fluorescence Imaging Probes: the fluorescent dye used for both NIR imaging and SPECT/CT, MRI.

Based on the principle of fluorescent probes, we divide the current fluorescent probes into four types and summarize them in **Figure 1**.

Most non-specific probes rely on a passive targeting strategy, which preferentially accumulates molecules in tumors. The passive targeting strategy attributes to the tumor microenvironment such as accumulation of acidity, anoxic habitat, and necrotic tissue (8). It is not specific so that burns, wounded, and other tissues can also retain more fluorescent dye than surrounding tissues.

The principle fluorescent component of tetracycline is achelate formed upon combination with calcium ions ontrabecular bone (9). OWEN et al. (10) studied the fluorescence of tetracycline medicines in bone cancers and normal bone in 1961. Normal bone tissue has strong fluorescence, while necrotic bone has no or low fluorescence. Tetracycline is nowadays used only infrequently for intraoperative imaging of bone and soft tissue sarcoma, not only because tetracycline drugs are toxic and have a high rate of adverse reactions, but also because the wavelength of tetracycline excitation light is 450-490nm, which is in the visible light range. This wavelength overlaps with normal tissues and is heavily absorbed in tissues such as hemoglobin and myoglobin (11).

Compared with fluorescent probes for fluorescence imaging in the visible region, the research direction in recent years has focused more on fluorescent probes with excitation wavelength in the near-infrared region NIR-1 (700-900nm). Near-infrared fluorescence with a wavelength of 700-900nm is rarely absorbed in tissues (12).

For example, the most commonly used and clinically approved fluorescent probe is indocyanine green (ICG). ICG has absorption and fluorescence spectra in the near-infrared (NIR) region. The excitation wavelength is 780nm, and it emits fluorescence in the range of 700-850nm. The red light is visible to the naked eye, but most of the light is not (13). Most researchers believe that the accumulation of ICG within solid tumors attributes to the EPR (enhanced permeability and retention) effect (14). Due to the presence of defective endothelial cells and wide fenestrations (600 to 800 nm) in nascent blood vessels, small molecules such as ICG are injected systemically and passively accumulate in tumors (15). However, Pandit et al. (16) pointed out that in addition to the EPR effect, transcytosis is the principle of molecular accumulation in tumors. It is the same as the research on ICG in Colorectal Cancer. Cancer cells have a high endocytic rate (17). ICG was preferentially taken up by cancer cells *via* clathrin-mediated endocytosis (CME) (18). Indocyanine green is a safe, basically non-toxic drug, which rarely reacts with other drugs (19). However, Indocyanine green accumulates in bone tumors, inflammation, and bone deformities.

Many factors can influence the EPR effect, including tumor type, size, and vascular mediators. As a result, the intensity of the ICG signal is unpredictable (20). If the patient has a fracture or ischemia at the surgical site during surgery, it will cause false-positive results and affect the judgment. According to research, encapsulation of ICG improves its targeting abilities and circulation time (21, 22).

### Activatable Fluorescence Probes

Some scientists have also designed activatable fluorescent probes that emit fluorescence only in tumor tissues. This probe contains a chemical group, which can be broken down *via* some enzymes in the tumor and microenvironment, therefore this probe is activated.

Many activatable fluorescent probes have applications in other types of tumors, and could theoretically be used for intraoperative imaging of bone and soft tissue tumors. The activatable fluorescent probes for the tumor microenvironment are mainly activated by extracellular enzymes specifically emitted in the tumor microenvironment. In addition to cathepsin-activated fluorescent probes that have been used in soft tissue sarcoma animal experimentation, there are also fluorescent probes activated by matrix metalloproteinases (23). The activatable fluorescent probes for tumor cells consist of two parts. One is the activation of intracellular enzymes, such as β-galactosidase bioactivation (24) and glutathione (GSH) bioactivation (25). And the other one is the activation of fluorescence by the tumor cell hypoxia environment (26). Besides, the pH of the tumor microenvironment is generally between 6.7-7.1, the pH of tumor cells is between 5.9-6.2, and the pH of advanced tumor cells can even reach 5.0-5.5, which is an acidic environment compared with normal tissues. Some probes are sensitive to pH, and their fluorescence is activated in an acidic environment allowing fluorescence imaging of tumors and tumor microenvironments (27).

Activatable fluorescence probes reduce the fluorescence intensity of normal tissues and further increase the tumor-to-background ratio (TBR). But at the same time, the chemical synthesis of probes is complicated, and there is still a lack of further research on the adverse reactions of these probes.

### Specific Fluorescent Probes

Unlike ICG with the EPR effect, specific probes do not rely on the tumor microenvironment but instead rely on a targeting moiety conjugated to a contrast agent with a high binding affinity. These probes have higher targeting properties than indocyanine green (28).

The original design method is to combine monoclonal antibodies with fluorescent dyes to create fluorescent probes. Previously, monoclonal antibodies were used as targeted drugs to treat tumors. For example, Bevacizumab is a monoclonal antibody that binds to vascular endothelial growth factor-A(VEGF-A)which is highly expressed in tumor cells (29) and plays a direct role in vascular endothelial production (30). Combine bevacizumab with the fluorescent dye IRDye800CW to synthesize a fluorescent probe that can specifically bind to tumors. Scientists designed Panitumumab-800CW (31) and Cetuximab-800CW (32) based on the principle of similars. Panitumumab is a monoclonal IgG2 antibody that binds to the Epidermal Growth Factor Receptor (EGFR) with high specificity (33). EGFR is highly expressed in bone and soft tissue sarcoma and is involved in osteolytic metastases of bone tumors. Cetuximab is also an anti-EGFR monoclonal antibody.

In recent years, with the development of chemical synthesis technology, moieties for active targeting have become available, such as nanoparticle scaffolds, peptides, ligands, and aptamers. Compared with antibodies, the moieties have similar binding characteristics but show better tumor penetration and more rapid clearance from non-targeted tissues (34). For example, ABY-029 is an EGFR-targeted affibody molecule labeled with IRDye 800CW (35). While performing intraoperative tumor fluorescence imaging, ABY-029 can be injected on the same day. Besides, compared with bevacizumab, panitumumab, and cetuximab, ABY-029 retains high EGFR specificity (36) with low immunogenicity and low toxicity (37).

Specific fluorescent probes are based on active targeting, their synthesis is complicated. Tumors are heterogeneous, so we can’t find a tumor marker expressed in each tumor tissue. The majority of specific probes are still in the pre-clinical stage. It requires more feasibility and toxicity studies, particularly for small molecule probes before clinical trials.

### Multimodality Fluorescence Imaging Probes

**Figure 2:**
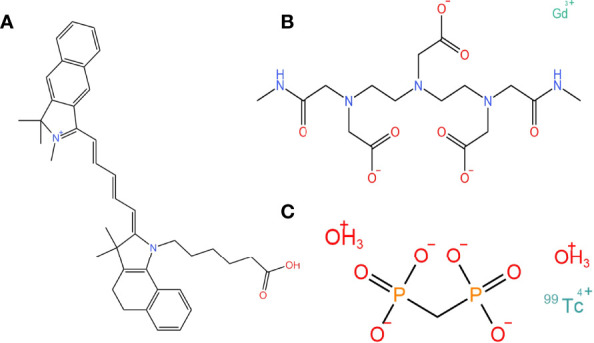
The chemical structure of fluorescence, magnetic, and SPECT nanoparticles that can compose multimodality probes. **(A)** A fluorescent dye: Cy5.5 carboxylic acid. **(B)** A magnetic nanoparticle as molecular imaging agent: gadodiamide. **(C)** A SPECT-CT tumor imaging agent: technetium Methylenediphosphonate(99mTc-MDT).

SPECT/CT, MRI, and NIR combined multimodal imaging technology have gained significant popularity. Scientists have designed fluorescent probes with SPECT/CT, MRI sensitive groups, and fluorescent dyes (38) (**Figure 2**). The contrast of preoperative SPECT/CT, MRI tumor imaging is improved by preoperative injection of multimodality fluorescent probes. The fluorescent sign of the tumor can also be collected during the operation. This combination of imaging and fluorescence imaging can significantly increase the detection rate of tumors and obtain more accurate tumor boundaries. This probe is used for preoperative tumor imaging, surgical planning, and intraoperative tumor fluorescence imaging.

Schematically, this kind of probe has much potential. For example, if we expand our scope to treatment, scientists have designed probes that combine Photodynamic therapy with fluorescence imaging (39, 40).

All imaging techniques have their limitations, e.g., MRI has problems with relatively low sensibility, and optical imaging has issues with low spatial resolution and small penetration depth (41). Multiple imaging techniques aid in early diagnosis and treatment planning. However, it is worth exploring whether it is necessary to enhance preoperative MRI and SPECT/CT tumor signal intensity in clinical practice (42).

In recent years, some researchers have focused on fluorescent probes in the NIR-2 range (1000-1700nm) and have produced several fluorescent probes for tumor imaging in the NIR-2 range (43). According to some researchers, fluorescence with a wavelength of 1000-1700nm, can reduce scattering when passing through the skin and is less affected by normal tissue autofluorescence. Compared with NIR-1 imaging, it can penetrate deeper tissues (44).

## Pre-Clinical Research

### Non-Specific Fluorescent Probes

Presently the widely used non-specific fluorescent probe in tumor surgery is indocyanine green. Overall, these cases in the past five years support that bone and tissue sarcoma can fluoresce after injection, and the tumor boundary is consistent with the pathological section control.

For example, Fourman (2018) (45) injected osteosarcoma cells into the left hind limb of BALB/c Mice. Researchers used pathological sections to confirm that the fluorescent part of the hind limb was a bone sarcoma. Mice with fluorescent lung tissue developed lung metastases from osteosarcoma. Interestingly, the researchers discovered that the higher the fluorescence intensity of the primary bone tumor, the greater the possibility of lung metastases. This finding suggests that we can early predict the probability of lung metastases in osteosarcoma patients through intraoperative bone tumor fluorescence intensity.

Another example of what is meant by Mahjoub (46), is that they injected ICG into 11 osteosarcoma mice 12 hours before surgery for fluorescence-guided osteosarcoma surgery. The recurrence rate of mice with osteosarcoma resection guided by ICG was much lower than that of mice with conventional resection.

In addition to indocyanine green, non-specific fluorescent probes such as Alizarin Red and Tetracycline had been expected to have great potential for intraoperative imaging of bone and soft tissue sarcoma. However, the fluorescence excitation wavelengths are 465nm and 490nm, which are both in the visible light range, and the fluorescence area overlaps with normal tissues. There have been few related studies in recent years.

### Activatable Fluorescence Probes

According to the different characteristics of tumor cells and tumor microenvironments from normal tissues, scientists have designed activatable optical probes. In 2016, Bartholf Dewitt S (47) used the cathepsin-activated fluorescent probe LUM015 in dogs with soft tissue sarcoma for intraoperative fluorescence imaging. According to previous studies, cathepsin is overexpressed in soft tissue sarcoma and other tumors while rarely expressed in normal tissues. All the dogs’ soft tissue sarcoma fluorescence when imaging. The pathologist took 33 parts of the excised tissue for biopsy, all of which were tumor tissues. The cathepsin-activated fluorescent probe is further exemplified in studies by Prince et al. (48). The researchers compare the TBR and effect of prosense750EX (another cathepsin-activated fluorescent probe) with multiple fluorescent probes for fluorescence imaging of soft tissue sarcoma. Prosense750EX, like the other probes in the study, can identify tumor beds with a diameter of less than 1mm intraoperatively. Therefore, the Prosense750EX can be used as a probe for fluorescence imaging.

### Specific Fluorescent Probes

Some specific probes have been designed, and animal experimentation has proved their specificity and sensitivity. Most specific probes are created by combining fluorescent dyes with antibodies or ligands that precisely bind to tumor cells (**Table 1**).

**Table 1:** Specific fluorescent probes for bone and soft tissue tumor imaging.

Target	Probe	Type of tumor	Name	Observation from postinjection	Year	author
CD105	Non-antibody-binding proteins	Osteosarcoma	A novel peptide targets CD105	1h	2018	Xiaolong Li (50)
EGFR	Affibody molecule	Synovial sarcoma	ABY-029	4h	2021	Hira Shahzad Sardar (52)
	Antibody	Fibrosarcoma	Cetuximab -IRDye800CW	9d	2018	Andrew C. Prince BSc (48)
EWS-FLI1	Peptide	Ewing sarcoma	CS2‐N‐E9R	6h	2021	Yu Wang (49)
integrin αvβ3	Small molecule	Fibrosarcoma	IntegriSense750	9d	2018	Andrew C. Prince BSc (48)
	Small molecule	Osteosarcoma	68Ga-CHS2	4h	2018	Yao Sun (73)
osteocalcin property receptor	Peptide	the lung metastases of osteosarcoma	CH1055-PEG -Affibody	12-36h	2020	Hui Zhou (51)
	Peptide	Osteosarcoma	CH1055-PEG-PT	12-36h	2020	Hui Zhou (51)
VEGFR2	Antibody	Fibrosarcoma	DC101 -IRDye800CW	7d	2018	Andrew C. Prince BSc (48)

Based on this probe design idea, our team designed a specific fluorescent probe CS2-N-E9R for Ewing’s sarcoma-specific fusion protein EWS-FLI1 (E/F) in 2021 (49). Our specific probe can make Ewing’s sarcoma fluorescence imaging in cell experimentation and animal experimentation. Besides, it does not show fluorescence for E/F-negative osteosarcoma cells.

For example, Li et al. (50) combined the non-antibody binding protein of CD105 with fluorescein isothiocyanate(FITC) to obtain a fluorescent probe targeting osteosarcoma. This fluorescent probe causes the osteosarcoma cells, dissected osteosarcoma tissues, and osteosarcoma in mice to emit fluorescence, proving that it can label osteosarcoma.

Another example of what is designed by Zhou (51) is CH1055-PEG-PT and CH1055-PEG-Affibody. These probes combined Small molecule protein binding to 143b osteosarcoma cells with Fluorescent dyes in the NIR-2 region. Both of these probes can image fluorescence in osteosarcoma. The surgeons used fluorescence guidance for tumor resection. Pathology specialists sectioned the tumor and adjacent tissues and stained them for microscopic examination after the surgery. The results revealed that the fluorescence intensity of the tumor was higher than that of adjacent tissues. The researcher suggests that, compared with CT, the fluorescent probe can image tumors smaller than 1 cm in diameter and has a clear fluorescence boundary.

This technology is further exemplified in animal experimentation using indocyanine green and ABY-029 combined fluorescence imaging in soft tissue sarcoma surgery. Sardar et al. (52) discovered that fluorescence imaging with ICG and ABY-029 is superior to ICG or ABY-029 alone. Among them, ABY-029 is more concentrated in the high-cell living tissue area, while ICG is more concentrated in the low-cell area. The article did not explore the reasons further. A possible explanation for these results may be related to the difference in imaging principles between the two probes. ICG accumulates more in new blood vessels, whereas ABY-029 binds to cancer cells specifically. It suggests that combining two fluorescent probes with different localization areas and fluorescence imaging principles could improve the specificity and sensitivity of fluorescence imaging in bone and soft tissue tumor surgery.

Xu’s experimentation study explored the feasibility of specific fluorescent probes used for intraoperative imaging after radiotherapy and chemotherapy (36). Xu designed a mouse model of soft tissue sarcoma after chemotherapy and radiotherapy and injected ABY-029 intraoperative fluorescence imaging into the mice 4-8 hours before surgery. It might be possible to estimate whether most patients with soft tissue sarcoma undergoing preoperative radiotherapy and chemotherapy can use ABY-029 Intraoperative fluorescence imaging. The results confirmed the feasibility of fluorescence imaging of soft tissue sarcoma in mice after radiotherapy and chemotherapy. This outcome is contrary to that of Nicoli et al. (53) who found indocyanine green could not fluorescently label osteosarcoma after radiotherapy. This result demonstrates the superiority of specific fluorescent probes compared to fluorescence imaging in indocyanine green.

Another research compared several fluorescent probes on soft tissue sarcoma mice (48). The researchers compare the intraoperative tissue fluorescence range with HE stained sections, and immunohistochemistry(IHC) to quantitatively compare TBR. Compared to DC101(binding to VEGFR-2) TBR 3.7, IntegriSense750(A small-molecule probe binding to integrin αvβ3) TBR 7.0, and ProSense750EX (activated by locally expressed cathepsin)TBR 5.8, the TBR of cetuximab-IRDye800CW was 16.8, which was significantly higher than other fluorescent probes.

In addition to the fluorescent probes that have been assessed on bone and soft tissue sarcoma, many newly designed fluorescent probes may have the potential to be used in intraoperative imaging of bone and soft tissue sarcoma. Mahalingam et al. (54) designed the Centyrin-Based Near-Infrared Probe, a fluorescent probe that images EGFR-positive tumors. Reviews show that osteosarcoma and soft tissue sarcoma can overexpress EGFR (55). In the future, we can build mouse models and conduct further animal experimentation to explore whether this probe is used for bone and soft tissue tumor imaging.

For a ligand or antibody that specifically binds to bone and soft tissue sarcoma, the ideal is to find a target not expressed in other tissues and expressed in all bone and soft tissue sarcoma, especially tissue cells surrounding the tumor. There are many studies on tumor-specific markers of bone and soft tissue tumor cells. CxCR4 (Cys-X-Cys receptor 4), PDGFR-β(Platelet-derived growth factor receptor-β), TEM1 (Tumor Endothelial Marker 1), VEGFR-1, EGFR, VEGFR-2, IGF-1R, IGF-2R, CD40, et al. are high specific tumor markers (56–58). Scientists use these tumor-specific markers to create antibodies or ligands and combine antibodies and ligands with fluorescent dyes to make specific fluorescent probes. According to animal experiments in the past five years, ligands and small-molecule peptides spread faster than antibodies and are more likely to accumulate in tumor tissue. There are numerous fluorescent dyes on the market currently, most of them are classified as rhodamines, oxazines, fluoresceins, cyanines, and carbopyronines in structure (59). The commonly used near-infrared fluorescent dyes such as IRDye800CW still have high development prospects.

In the case of specific fluorescent probes, future research could focus on developing new probes specifically binding to bone and soft tissue sarcoma, determining whether existing fluorescent probes can be used for bone and soft tissue sarcoma, and evaluating the advantages, disadvantages, and effectiveness of the probes.

### Multimodality Fluorescence Imaging Probes

Probes for multimodal visualization in MRI, SPECT/CT, and Near-Infrared Optical Imaging have gotten attention in the past five years. These probes have the potential for preoperative tumor imaging, surgical planning, and intraoperative tumor fluorescence imaging.

It is exemplified in the animal experimentation undertaken by Xu with 99mTc-Gd@OVA-Cy nanoprobe (60). Researchers performed preoperative NIR fluorescence imaging, MRI, and SPECT/CT of osteosarcoma with nanoprobe. After 15 minutes of intravenous injection of the fluorescent probe, the images of all three modes showed enhanced signals of osteosarcoma. In MRI, SPECT/CT, and NIR imaging, researchers can observe a clear boundary of osteosarcoma, and the tumor boundary is consistent with the results of HE staining sections. Surprisingly, the researchers also found that the fluorescent probe can show lymph drainage and sentinel lymph nodes. Therefore Xu considered that this probe might be used for osteosarcoma to improve lymph node resection and preoperative planning.

Scientists designed many fluorescent probes for multimodal imaging in the past five years. But there are few animal experiments on whether these fluorescent probes can be applied to bone and soft tissue sarcoma. Lee et al. (61) designed an Nd3+-UCNPs nanoprobe specifically binding to CD44. The nanoprobe is injected into the hepatocellular carcinoma of patients, used for preoperative MRI detection and intraoperative NIR tumor imaging. Related literature shows that bone and soft tissue sarcoma can express CD44 (62). Therefore, this multimodal probe may be significant in intraoperative and preoperative tumor imaging for CD44-positive bone and soft tissue sarcoma.

Researchers also focus on probes for multimodal visualization in SPECT/CT and intraoperative near-infrared optical imaging. A notable example is the folate-ECG-ROX targeted folate receptor in the tumor (63). Another example designed by Manca is the ICG-99mTc probe, which facilitates visualization of lymph drainage and assesses the sentinel lymph node (64).

### Clinical Trials

Reports about intraoperative fluorescence imaging of bone and soft tissue sarcoma are limited (**Table 2**). In 2019, Samkoe et al. (65) reported a case of using ABY-029 intraoperative fluorescence imaging for soft tissue sarcoma. The intraoperative fluorescence intensity ratio of soft tissue sarcoma to normal tissue/background is 2.0/3.4, which is sufficient to distinguish tumor from normal tissue by fluorescence during operation. The tumor was stained with hematoxylin-eosin staining and IHC postoperatively, and the fluorescent tissue was confirmed to be soft tissue sarcoma, and the fluorescence signal was highly associated with the expression of EGFR.

**Table 2:** Clinical Trials in fluorescence imaging for bone and soft tissue sarcoma surgery.

	Author	Year	Study type	Studypopulation	Fluorescentpopulation	Type of probe	Target	Type of tumor	Interval betweeninjection andsurgery
ICG	Nicoli, Fabio MD (53)	2021	Prospective study	10	8	Non-specific	.	Osteosarcoma(0/1) Chondrosarcoma(1/1) Myxofibrosarcoma(4/5) Pleomorphic sarcoma(1/1) Leiomyosarcoma(1/1) Myxofibrosarcoma(1/1)	16-24h
ICG	Jarrod D Predina (67)	2019	Phase 1 clinical trial	30	30	Non-specific	.	Lung metastases of bone and soft tissue sarcoma	24h
5-ALA	Florian Scheichel (68)	2020	Retrospective study	11	11	Non-specific	.	Bone-infiltrating meningiomas	3h
OTL38	Jarrod D. Predina (69)	2018	Case report	1	1	Specific	FR-α	Lung metastases of osteosarcoma	4h
Bevacizumab -800CW	Pieter J Steinkamp (66)	2021	Phase 1 clinical trial	15	15	Specific	VEGF-A	Myxofibrosarcoma(7/7) Liposarcoma(3/3) Synovial sarcoma(2/2) Leiomyosarcoma(1/1) Angiosarcoma(1/1) Undifferentiated pleomorphic sarcoma(1/1)	3d
ABY-029	Kimberley S. Samkoe (65)	2019	Case report	1	1	Specific	EGFR affibody	Pleomorphic sarcoma(1/1)	1-3h

In a similar case in the UK, 11 patients with bone and soft tissue sarcoma were admitted for ICG intraoperative fluorescence imaging (53). ICG was injected intravenously 16-24 hours before the operation, and the Stryker Spy Phi near-infrared device collected the fluorescence signal during the operation. Surgeons believe that in three of the 11 cases, they removed more tissue during the operation due to fluorescence. Nine of the 11 instances revealed tumor fluorescence during surgery. Two instances exhibited no fluorescence during surgery, one was grade 1 myxofibrosarcoma, and the other was osteosarcoma with more than 90% necrosis after chemotherapy. The failure could be because ICG fluorescence imaging is better suited to tumors with a higher degree of malignancy, no treatment, and fewer necrotic areas.

This technology is further demonstrated in studies using Bevacizumab-IRDye800cw fluorescence imaging in 15 patients with soft tissue sarcoma during surgery (66). Researchers found fluorescence in soft tissue sarcoma during and after the operation in all 15 cases and no adverse reactions. Furthermore, the researcher discovered that the necrotic area of soft tissue sarcoma treated by neoadjuvant chemotherapy had no fluorescence. Auspiciously, we noticed in clinical practice that the necrotic area is more inside the tumor and has few effects on the fluorescence of the tumor border.

Furthermore, bone and soft tissue tumor metastasis are frequent. Fluorescence imaging can detect tumor metastasis in bone and soft tissue. These clinical trials reveal the need for fluorescence imaging among metastases. Patients subjected toI CG injection were assessed after 24 hours (not overlapping with the optimal time for ICG to show bone and soft tissue sarcoma). Among 44 patients with soft tissue sarcoma lung metastases, 40 lung metastases showed fluorescence during Video-assisted thoracoscopic surgery(VATS). Among 40 cases of osteosarcoma lung metastases, 36 cases had fluorescence. The depth of all lung metastases without fluorescence imaging was more than 2 cm. According to Predina, fluorescence imaging during ICG surgery is better for detecting tumor metastasis with a depth smaller than 2 cm and a diameter greater than 5 mm (67).

Scheichel (68) performed a clinical trial using 5-aminolevulinic acid (5-ALA) intraoperative fluorescence imaging in fifty patients with bone and soft tissue infiltrating meningiomas. All bone fluorescence shows tumor invasion into bone tissue. Three patients showed additional fluorescence in the periosteum and temporal muscles, and histopathological examination confirmed tumor infiltration (68).

Predina and colleagues studied a patient with osteosarcoma lung metastases undergoing surgery and showed that fluorescence imaging with OTL38 enabled the detection of Lung metastases. According to previous studies, FR-α is overexpressed in 80% of primary osteosarcoma. The lung metastases had strong fluorescence after intravenous injection of 0.025mg/kg OTL38. However, the researchers did not specify whether fluorescence was observed in the primary osteosarcoma (69).

### Future Perspectives

At present, intraoperative fluorescence imaging does not use quantitative norms to determine whether it is tumor tissue. There is no standard for how high the fluorescence contrast should be to indicate a tumor in intraoperative fluorescence imaging technology. To determine the standard, it is important to conduct clinical trials including large sample size and compare with pathological results. Futhermore, a technique combining biophysics-inspired modeling and artificial intelligence (AI) was envisioned to monitor intraoperative changes in NIR intensities over time in different tissue and provide clinically significant lesion identification (70). In addition, mixed reality(MR) techniques that combine fluorescence imaging with CT have been used in liver resection (71). We can embed an augmented reality (AR)-based navigation system in the fluorescence imaging devices (72), and evaluate the usefulness of the system in the experimental study.

The manufacture and use of fluorescent probes for intraoperative fluorescence imaging of bone and soft tissue sarcoma have a potential future. Non-specific probes may additionally fluoresce in non-tumor areas, which can cause surgeons to misjudge. The main direction of new fluorescent probes will be specific fluorescent probes with high specificity to label tumors. With the further investigation of the mechanism of bone and soft tissue sarcoma, scientists will discover more specific tumor-expressed molecules. We can accordingly design specific fluorescent probes with high specificity and sensitivity.

Simultaneously, we noticed that tumors are heterogeneous, and it is difficult for a probe to image all tumors of the same type. Experiments are currently underway to combine two fluorescent probes with different principles to increase accuracy and lower the negative rate. In the future, we can design fluorescent probes with multiple responses to tumors and the microenvironment to further reduce the false-negative rate of fluorescence during tumor surgery.

## Author Contributions

ZC: writing-original draft. HH: visualization, formal analysis. SH: investigation. YW: supervision. LC: conceptualization. YX: writing—review and editing. All authors contributed to the article and approved the submitted version.

## Funding

This work was supported by National Natural Science Foundation of China (NO. 82103285), the Improvement Project for Theranostic ability on Difficulty miscellaneous disease(Tumor) (ZLYNXM202005), the Research Fund from Medical Sci-Tech Innovation Platform of Zhongnan Hospital, Wuhan University (PTXM2021003).

## Conflict of Interest

The authors declare that the research was conducted in the absence of any commercial or financial relationships that could be construed as a potential conflict of interest.

## Publisher’s Note

All claims expressed in this article are solely those of the authors and do not necessarily represent those of their affiliated organizations, or those of the publisher, the editors and the reviewers. Any product that may be evaluated in this article, or claim that may be made by its manufacturer, is not guaranteed or endorsed by the publisher.

## Abbreviations

CT, computed tomography; MRI, magnetic resonance imaging; SPECT, single photon emission computed tomography; TBR, tumor-to-background ratio; ICG, indocyanine green; NIR, near-infrared; IHC, immunohistochemistry; VEGF, vascular endothelial growth factor; EGFR, epidermal growth factor receptor; VEGFR, vascular endothelial growth factor receptor; 5-ALA, 5-aminolevulinic acid; FR-α, folate receptor-α; insulin-like growth factor, IGF; EPR, enhanced permeability and retention; CME, clathrin-mediated endocytosis; glutathione, GSH.
